# Modulation of test anxiety-induced salivary protein secretion by ovarian steroid hormones: a preliminary study

**DOI:** 10.1007/s13105-025-01067-w

**Published:** 2025-02-08

**Authors:** Lorenzo Zallocco, Maurizio Ronci, Andrea Pantalone, Maria Rosa Mazzoni, Eleonora Ramoretti, Antonio Lucacchini, Laura Giusti, Laura Sebastiani

**Affiliations:** 1 Department of Translational Research and New Technologies in Medicine and Surgery, University of Pisa, Via San Zeno 31, Pisa, 56123 Italy; 2 Department of Medical, Oral and Biotechnological Sciences, University “G. D’Annunzio” of Chieti-Pescara, Chieti, Italy; 3 Interuniversitary Consortium for Engineering and Medicine, COIIM, Campobasso, Italy; 4 Department of Medicine and Science of Aging, Clinic of Orthopaedics and Traumatology, University “G. D’Annunzio” of Chieti-Pescara, Chieti, Italy; 5 Department of Pharmacy, University of Pisa, Pisa, Italy; 6 School of Pharmacy, University of Camerino, Camerino, Italy; 7 Department of Clinical and Experimental Medicine, University of Pisa, Pisa, Italy

**Keywords:** Ovarian steroid hormones, Test anxiety, Saliva, Proteomics, Stress-related proteins, Oral mucosa immunity

## Abstract

In women the menstrual cycle influences mood and anxiety. Aim of this study was to preliminarily investigate whether different ovarian steroid hormone levels may modulate the psychophysiological responses elicited by test anxiety. Specifically, we compared the secretion of anxiety-induced salivary proteins of healthy women in the early follicular (Pre-Ov group) (low ovarian steroid hormones levels) and mid-luteal (Post-Ov group) (medium/high ovarian steroid hormones levels) phase of the menstrual cycle, during the simulation of an oral examination. Saliva samples were collected before and after a relaxation period and at two post-simulation times and analyzed by two-dimensional electrophoresis and western blot. Proteins corresponding to spots differentially expressed in the two groups across the session were identified through mass spectrometry and most of them corresponded to acute stress and/or oral mucosa immunity biomarkers. The task induced an increase in alpha-amylase, carbonic anhydrase and cystatin S, and a decrease in immunoglobulin light/J chains in both groups. Analogous changes in these proteins have previously been linked to psychological or physical stress. However, specific spots corresponding, for example, to cystatins and 14-3-3 protein, changed exclusively in the Pre-Ov group, while prolactin-inducible protein, polymeric immunoglobulin receptor, fragments of alpha-amylase and immunoglobulins only in the Post-Ov group, indicating a potential modulation of their secretion by ovarian steroid hormones. Overall, the results provide preliminary evidence that ovarian steroid hormones may be a driving factor for differences in physiological responses induced by test anxiety. The results are promising, but further validation in a larger sample is needed.

**Supplementary Information:**

The online version contains supplementary material available at 10.1007/s13105-025-01067-w.

## Key points

● Text anxiety prompts changes in the secretion of various salivary proteins.

● We studied how these protein changes relate to ovarian steroid hormone levels.

● Many anxiety-modulated proteins were exclusive to women with high hormone levels.

● Ovarian steroid hormones modulate the physiological biomarkers of test anxiety.

**Supplementary Information:**

The online version contains supplementary material available at 10.1007/s13105-025-01067-w.

## Introduction

Mood disorders affect more women than men and ovarian steroid hormones (OH) are likely to contribute to the observed gender differences. Gender differences in anxiety begin to emerge at puberty and in women, negative mood and the risk of worsening anxiety symptoms increase during low estradiol phases of the menstrual cycle and during the perimenopausal period characterised by decreased sex hormone levels [1]. Also, some forms of depression are specific to women, including pubertal depression and premenstrual dysphoric disorder [2–4] while the distress reported following a stressful event is inversely correlated with estradiol levels within the same menstrual cycle phase [1]. Mood/stress regulation can be attributed to the capacity of OH to cross the blood-brain barrier and to the existence of several OH receptors within brain areas involved in emotional processes [5–7].

In a previous study, we showed that test anxiety, defined as a multidimensional construct characterized by a set of psychophysiological manifestations that accompany concern about possible negative consequences at an examination [8], was able to modify the expression of salivary proteins considered as typical markers of stress and anxiety (e.g. α-amylase). The expression of other salivary proteins involved in humoral immune and inflammatory responses, such as cystatin, which appear to be regulated by OH (e.g., β-estradiol is an upstream regulator of cystatin), was also found to be altered [9]. In addition, a greater variability in the expression of immunoglobulins light chains in females than in males was observed (unpublished observation).

Given these premises, we hypothesized that there could be differences in anxiety-induced salivary protein expression between women experiencing test anxiety in different phases of the menstrual cycle that are characterized by different OH levels. Thus, the present study was designed as a preliminary investigation aimed at exploring whether different levels of OH may modulate salivary protein secretion prompted by the same test anxiety task used by Zallocco et al. [9], namely a simulation of an oral examination. More specifically, we investigated the potential impact of the examination simulation in two groups of young, healthy females, one in the early follicular phase (low OH levels) and the other in the mid-luteal phase (medium/high OH levels) of the menstrual cycle. In addition to the collection of saliva samples, EEG, heart rate and perceived anxiety levels were also measured in order to test the effectiveness of the task in inducing anxiety but also to explore the possible interaction among OH, EEG, heart activity and perceived test anxiety.

## Methods

### Participants

The study was performed in accordance with the Declaration of Helsinki ethical standards and approved by the Committee on Bioethics of the University of Pisa (Review No. 5/2018, 30th November 2018). All participants read and signed an informed consent.

Twenty-five healthy female students from the University of Pisa aged 23.4 ± 2.84 years (mean ± SD), volunteered to take part in the study. Females had to be regularly menstruating (i.e., every 26–30 days over the previous semester) and not taking contraceptives within the past 3 months. Moreover, participants could not be taking medications nor having prior history of neurological, psychiatric, or medical diseases. The presence of such disturbances or the use of medication could indeed influence the set of psychophysiological manifestations of test anxiety to some degree. Females that have been pregnant within the past year or lactated within the past 6 months were also excluded. Finally, only participants with no dental or periodontal problems were included in the study. Indeed, participants underwent a dental/periodontal screening within 15 days of the experiment.

Each participant was administered a battery of self-report questionnaires to establish an individual profile of general and social/test anxiety and exclude the presence of social phobia. The following scales were administered: the Leibowitz Social Phobia Scale [10], the Trait Anxiety Inventory [11], the Social Phobia Scale, the Social Interaction Anxiety Scale [12], the Italian Social Phobia Inventory [13], the Brief Social Phobia Scale [14], the Hamilton Anxiety Scale [15], the Perceived Stress Scale [16], and the Westside Test Anxiety Scale [17].

Participants took part at the experimental session either at the 3rd day of the menstrual cycle (early follicular phase), or at the 21st day of the cycle, after the ovulatory phase. Accordingly, we divided participants into the Pre-Ov (*n* = 11) and the Post-Ov group (*n* = 14).

### Experimental protocol

The experiments were scheduled at 9:00–10:00 a.m. Participants were asked to abstain from eating, drinking coffee or juices, smoking, chewing gum or candies, brushing their teeth, and using lipstick for a minimum of 3 h before the experimental session. Upon arrival, participants were informed of the experimental procedures and provided written informed consent. Subsequently, the first saliva sample (T1) was collected.

They were then equipped with the g-Nautilus 32 system (gTech, Schiedlberg, Austria) to record synchronized EEG and ECG signals (see Catrambone et al. 2024 for recording details [18]). For ECG recording, 2 Ag/AgCl disposable electrodes were placed on the chest, according to ECG lead I, and connected to the EEG headset. Signals were acquired by means of the g.Recorder software.and ECG was analysed by means of Kubios HRV Premium software in order to obtain the series of the distances (msec) between consecutive R waves (RR). The experimental protocol was the same as in previous studies [9, 18]. To begin, the subject was instructed to engage in a 10-minute session of closed eye relaxation. The subject was seated in a semi-reclined armchair and was provided with headphones through which the sound of sea waves could be heard. The subject was instructed to keep their eyes closed, relax, and breathe at their usual pace. At the end of this session participants gave a second saliva sample (T2) and completed the STAI-Y1 questionnaire on anxiety state [11]. The subsequent phase consisted of a test anxiety task, which comprised three consecutive stages: a 2-minute open-eye phase, a study phase and oral examination phase. During the study phase, the participant was seated in front of a computer screen where a written text was presented for a period of three minutes, during which they were asked to read the text and memorise it. Throughout this phase, the elapsed time was indicated on the computer screen. At the end of the study phase, participants completed the Italian version of the Speech Preparation Questionnaire (PREP) [19] aimed to assess their confidence, nervousness, calmness, and preparedness before the oral presentation and to predict the quality of their performance. The examination phase consisted of the oral presentation of the text to a role-playing “professor”, who would evaluate their performance on a 30-point scale (the same utilized by the Italian university to score examinations). The session lasted two minutes or until the participant stated that they did not remember any further information. Another saliva sample was collected at the end of the test anxiety task (T3), and the STAI-Y1 was completed again. Finally, participants were interviewed regarding the experienced feelings and after 20 min from the task end, a last saliva sample was taken (T4).

### Whole saliva collection

Whole saliva (WS) was obtained through spitting from each participant at T1, T2, T3 and T4. In order to overcome possible differences in flow rate, we collected the same amount of saliva (1 ml) for all participants. After collection, WS samples were immediately centrifuged at 16,000 g for 20 min at 4 °C to yield clear samples. The protein concentration in the resulting supernatants was measured by Bio-Rad DC protein assay (Bio-Rad, Hercules, CA, USA) using bovine serum albumin as the reference standard. Samples were stored at -80 °C until use.

### Enzyme-linked immunosorbent assay

WS levels of β-estradiol and progesterone were detected using ELISA kits following the manufacturer’s instructions. The β-estradiol ELISA kit was from Demeditec (Kiel, Germany, cod. DESLV4188) while the progesterone kit was from Enzo Life Science (New York, USA, cod. ADI-900-011). The sensitivity of β-estradiol and progesterone assays was 0.6 pg/mL and 8.5 pg/mL, respectively.

### Two-dimensional electrophoresis

WS samples at T2, T3 and T4 from 20 participants (*n* = 10 Pre-Ov; *n* = 10 Post-Ov) were analysed by 2-DE according to Zallocco et al., 2021 [9]. Briefly, 200 µg of salivary proteins were loaded and separated on 18 cm Immobiline Dry Strips with a linear pH gradient of 3–10 (Serva, Heidelberg, Germany) for the first dimension and then the second dimension was performed on 11-16.5% gradient polyacrylamide gels. The gels were then stained with 1 µM Ruthenium II tris (bathophenanthroline disulfonate) tetrasodium salt (RuBP) (Cyanagen, Bologna, Italy) according to Lamanda et al. [20] and resulting images were acquired using the ImageQuant LAS4010 (GE Health Care, Chicago, IL, USA). Spots found differentially expressed across the task were excised from the gels and processed for identification by mass spectrometry.

### In-Gel digestion and mass spectrometry

The gel pieces were processed according to Ciregia et al. [21]. Resulting peptides were identified as previously described [22] using an UltiMate3000 RSLCnano chromatographic system coupled to an Orbitrap Fusion Tribrid mass spectrometer (Thermo Fisher Scientific, Waltham, MA, USA) operating in positive ionization mode, equipped with a nanoESI source (EASY-Spray NG). Raw data were directly loaded in PEAKS Studio Xpro software (Bioinformatic Solutions Inc, Waterloo, Canada) using the ‘correct precursor only’ option. The mass lists were searched against the Uniprot/SwissProt database (downloaded June 2021) to which a list of common contaminants was appended, selecting human taxonomy (20,619 searched entries). Non-specific cleavage was allowed to one end of the peptides, with a maximum of 2 missed cleavages and 2 variable PTMs per peptide. Ten ppm and 0.5 Da were set as the highest error mass tolerances for precursors and fragments, respectively. -10lgP threshold for PSMs was manually set at 35.

### Western blot analysis

To validate the difference of immunoglobulin α-chain C (IGHA1) and α-amylase 1 (AMY1) expression observed between groups at different times of stress task, western blot (WB) analysis was performed. Aliquots of salivary proteins (3 and 5 µg for AMY1 and IGHA1, respectively) of 12 subjects (6 Pre-Ov and 6 Post-Ov) were analysed as previously described [9].

Primary antibodies were diluted as follow: 1:500 dilution of anti-IGHA1 polyclonal antibodies (#PA5-14361, Thermo Fisher Scientific, Waltham, MA, USA) and 1:1000 dilution of anti-AMY1 rabbit monoclonal antibodies (#3796, Cell Signalling Technology, Danvers, MA, USA). Immuno-labelled bands were analysed with the Image Quant-TL software, normalizing the optical density of each band to the total protein optical density obtained with RuBP staining [9, 21].

Two-dimensional gel electrophoresis coupled to WB was employed to validate the presence of α -amylase fragments. Two-dimensional blots were performed essentially as previously described [23] using the same anti-AMY1 rabbit monoclonal antibody mentioned above.

### Bioinformatic analysis

The significant proteins retrieved from comparative analysis of Pre-Ov vs. Post-Ov group were analysed using Ingenuity Pathway Analysis (IPA) software (QIAGEN Redwood City, USA, www.qiagen.com/ingenuity, Build version: 321501 M Content version: 21249400) that enables the determination of predominant canonical pathways and involved interaction networks. Each protein identifier, the corresponding ratio value (T3/T2, T4/T2, and T4/T3), and *p*-value were entered into the software for comparative analysis. Such analysis highlighted upstream regulator whose activity appears to change significantly based on the *z*-score value in the two groups under examination.

### Statistical analysis

Data analysis was performed by means of GraphPad Prism version 8 (GraphPad Software, CA, USA)and SPSS Statistics software (IBM, Chicago, IL, USA) and Same Spot Software (v4.1, Total Lab, Newcastle Upon Tyne, UK). Data normal distribution was assessed through Shapiro-Wilk test.

STAI-Y1 scores were analysed by means of repeated measures ANOVA (ANOVArm) with Task (T2 and T3) and Group (Pre-Ov and Post-Ov) as within and between subject effects, respectively, while PREP scores of the two groups on the five self-report items were analysed through MANOVA.

RR data were analysed by ANOVArm with Phase (relaxation, study, and examination) and Group (Pre-Ov and Post-Ov) as within and between subject effects, respectively.

Analysis of proteomic data within each group was carried out by ANOVArm with Task (T2, T3, and T4) as within subject factor while comparison between groups at T2 by unpaired Student’s *t*-test.

For ELISA and WB data, comparisons between the two groups were performed by means of unpaired Student’s *t*-test. The cutoff for statistical significance was *p* < 0.05 and power > 70%. For multiple-comparisons correction the FDR adjusted p-value (q-value) was applied. Q-value cutoff was set at *q* < 0.05; q-value up to 0.1 were also accepted only if power was > 90%.

In both groups, Pearson correlation analysis between progesterone and estradiol normalized values and both the optical density of protein spots at T2 and the ratio values of T3/T2, and T4/T2 was carried out. Cut off for correlations was set at *r* > 0.45 or < -0.45.

## Results

### Questionnaires

The scores of the two groups in the STAI-Y2, LSPS, SPS, SIAS, WTAS, I-SPIN, BSPS, HAS and IPSS-10 are shown in Table 1. A comparison of the two groups’ scores on the different tests (unpaired t-test) showed no significant difference between the two groups for either general or social/test anxiety.

Analysis of STAI-Y1 scores revealed a significant Task effect (F (1.23) = 61.418, *p*-value < 0.001) with scores at T3 higher (47.96 ± 10.63) than at T2 (32.80 ± 5.99). No significant group differences nor interactions were found. Concerning PREP results (Table 1), significant higher scores in the Post-Ov than Pre-Ov group were found for Confidence, Preparedness and Performance Goodness Prediction.

**Table 1:** Questionnaires for evaluating psychological profile of participants

Scales	Group		Unpaired t test			
	Pre-Ov (*n* = 11)	Post-Ov (*n* = 14)	t	*p*-value		
Liebowitz total	52.73 ± 29.69	40.14 ± 17.11	1.333	0.196		
*Liebowitz fear/anxiety*	28.82 ± 15.40	24.28 ± 10.58	0.872	0.392		
*Liebowitz avoidance*	23.90 ± 16.11	15.86 ± 8.49	1.612	0.121		
Westside	2.84 ± 0.79	2.71 ± 0.66	0.450	0.657		
I-SPIN	29.00 ± 17.06	20.35 ± 11.63	1.506	0.146		
BSPS total	19.45 ± 12.00	19.57 ± 8.20	0.029	0.977		
*BSPS -Fear*	7.27 ± 3.98	6.92 ± 3.52	0.229	0.821		
*BSPS -Avoidance*	6.27 ± 4.60	6.71 ± 2.87	0.294	0.771		
*BSPS -Physiology*	5.91 ± 4.53	5.93 ± 3.22	0.012	0.990		
STAI-Y2	50.36 ± 12.31	52.14 ± 10.61	0.388	0.702		
SPS	28.82 ± 19.90	24.64 ± 15.05	0.598	0.556		
SIAS	27.36 ± 18.11	27.36 ± 8.79	0.001	0.999		
HAS	24.00 ± 12.31	20.73 ± 11.20	0.673	0.508		
IPSS-10	25.09 ± 8.82	25.54 ± 10.36	0.113	0.911		
PREP	**Group**		**MANOVA**			
	**Pre-Ov (n = 11)**	**Post-Ov (n = 14)**	**F(1, 24)**	**p-value**		
Nervousness	3.20 ± 1.40	3.64 ± 1.08	0.798	0.196		
Confidence	1.50 ± 0.81	2.36 ± 1.08	4.794	0.039		
Calmness	1.70 ± 0.78	1.93 ± 0.92	0.435	0.516		
Preparedness	1.40 ± 0.66	2.42 ± 1.02	8.407	0.008		
Performance Goodness Prediction	1.30 ± 0.64	2.21 ± 0.89	8.192	0.009		

### Heart beats interval (RR) analysis

Analysis of RR data yielded a significant Phase effect (F (1.22) = 8.22) *p*-value = 0.001, η^2^ = 0.31), with lower values during the oral examination (0.670 ± 0.144 ms; mean ± SD) than during the relaxation phase (0.824 ± 0.162 ms). No significant Group effect nor interaction were found.

### Ovarian hormone levels

**Fig. 1:**
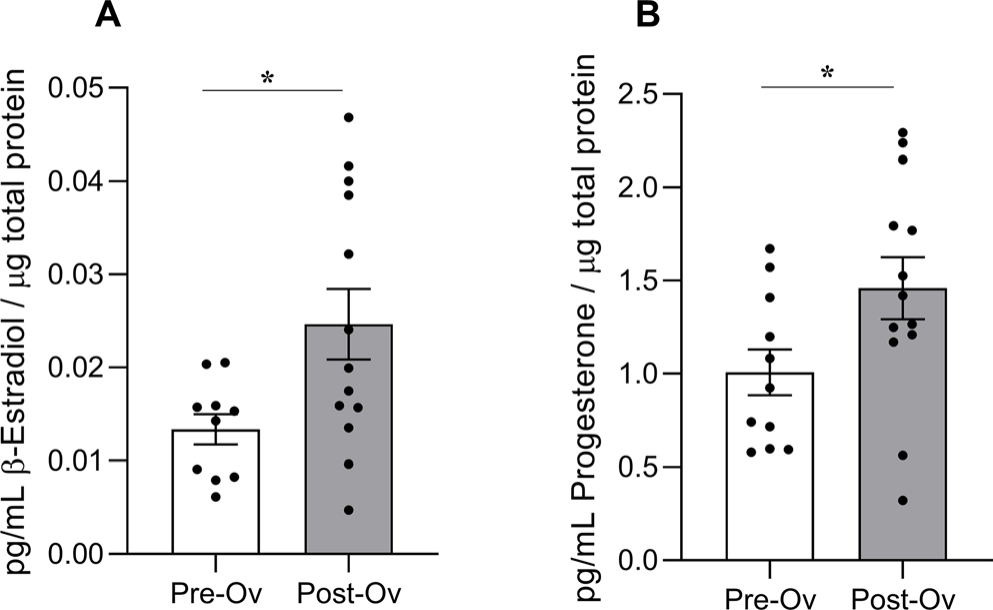
Histograms of the normalized concentration of (**A**) β-estradiol and (**B**) progesterone in the Pre-Ov and Post-Ov group. The bar graph shows the mean ± SD of the hormone concentrations normalized on total salivary proteins. An unpaired *t*-test was used to compare differences among groups with significance assessed at *p*-value < 0.05

The OH concentration values in the Pre-Ov and Post-Ov group were 0.0134 ± 0.0052 and 0.0246 ± 0.0137 for β-estradiol and 1.008 ± 0.406 and 1.459 ± 0.600 for progesterone, respectively. According to the trend of menstrual cycle, significant higher levels of both gonadal hormones were observed in the Post-Ov than Pre-Ov group (progesterone: t = 2.114, df = 22, *p*-value = 0.0461; β-estradiol: t = 2.464, df = 22, *p*-value = 0.0224) (Fig. 1).

### Proteomic analysis

A proteomic approach was employed to evaluate potential expression changes of salivary proteins occurring at different phases of the test anxiety task. All participants were able to provide 1 ml of saliva in about 1 min at each of the three sampling times. Salivary samples collected at T2, T3, and T4 for Pre-Ov and Post-Ov groups were processed by 2-DE (Figure S1 and S2). Comparative analysis of 2-DE images at post-relaxation time (T2) highlighted 16 differentially expressed protein spots between the Pre-Ov and Post-Ov group. A list of identified proteins is shown in Table S1. Among these proteins, a 1.34 to 1.59-fold increase of immunoglobulin λ and κ light chains (IGLC/IGKC) and J chain (JCHAIN) was observed in WS of Post-Ov subjects with respect to Pre-Ov subjects. Moreover, a 1.8-fold increase of profilin 1 (PFN1) and a 1.4- to 2.1-fold decrease of AMY1 were found. A 1.47-fold decrease of transketolase (TKT) and 1.49-fold of arginase1 (ARG1) in the Post-Ov group compared to the Pre-Ov group was also detected. However, as indicated by the high q-value (Table S1), none of these differences survived the multiple-comparisons correction.

**Fig. 2:**
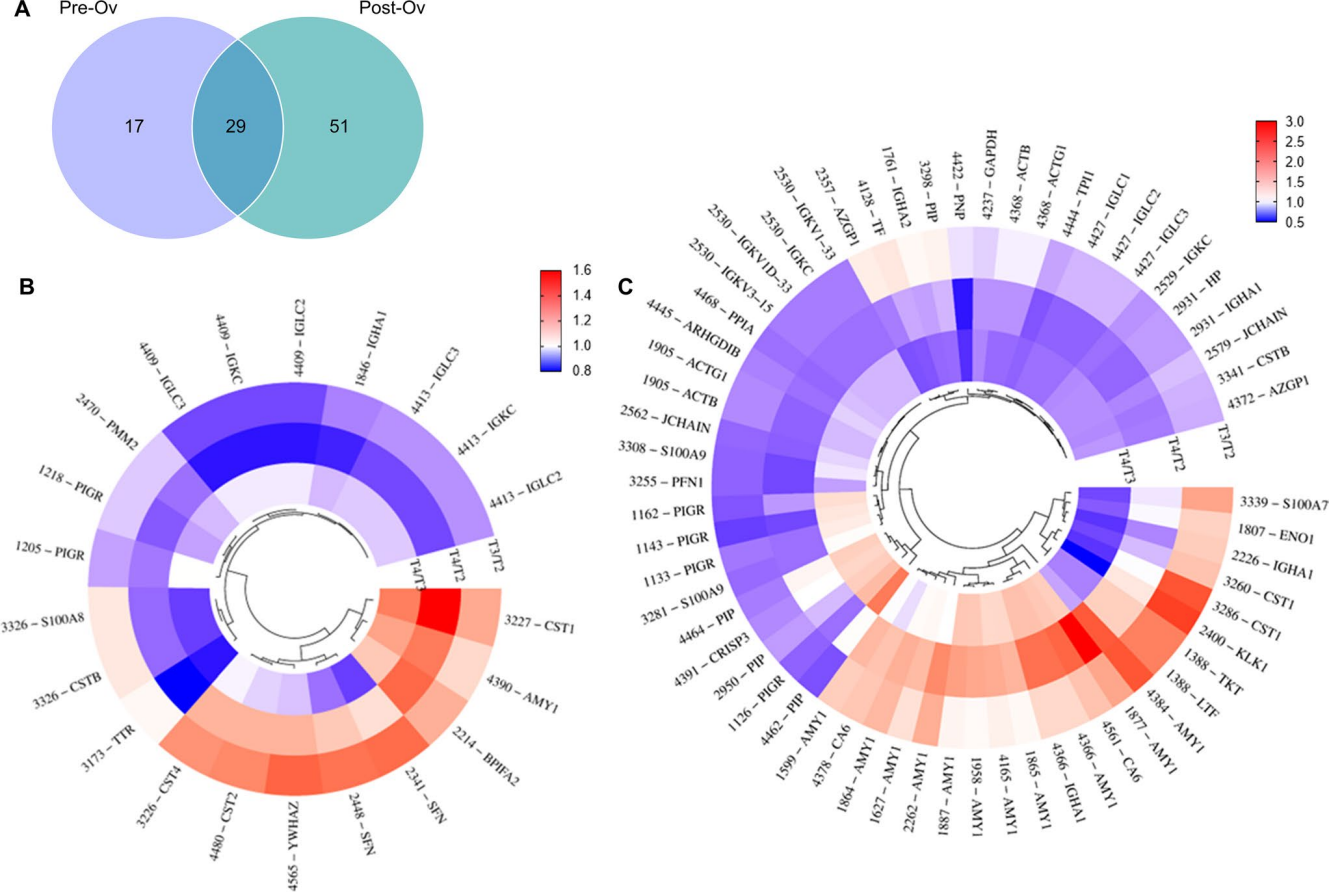
Significant differentially expressed proteins according to statistical analysis. (**A**) Venn diagram showing the number of protein spots found differentially expressed using ANOVArm in the Pre-Ov and Post-Ov group. (**B**) Circular cluster heatmap of differentially expressed protein spots exclusive of the Pre-Ov group. (**C**) Circular cluster heatmap of differentially expressed protein spots exclusive of the Post-Ov group. Red and blue shadings represent higher and lower relative expression levels, respectively

Forty-six and eighty protein spots showed significant expression changes across the task as revealed by ANOVA analysis individually conducted on the Pre-Ov and Post-Ov group, respectively (Fig. 2A). Seventeen and fifty-one protein spots were exclusive of the Pre-Ov and Post-Ov group, respectively, whereas 29 were in common. Among the latter, AMY1 (7 protein spots), and immunoglobulin subunits (12 protein spots of light chains and J chain), whose secretion increased and decreased, respectively, could be linked to the acute stress condition rather than ovarian hormonal influence. Considering other common spots, carbonic anhydrase 6 (CA6) and cystatin-S (CST4) deserve to be mentioned since these proteins have been associated with acute stress [25]. Specifically, CA6 increased across the task (i.e. T4 > T3 > T2), while CST4 exhibited an increase between T2 and T3, followed by a decrease after twenty minutes (T4). In Table S2, the complete list of common differentially expressed proteins at different task times is shown.

**Fig. 3:**
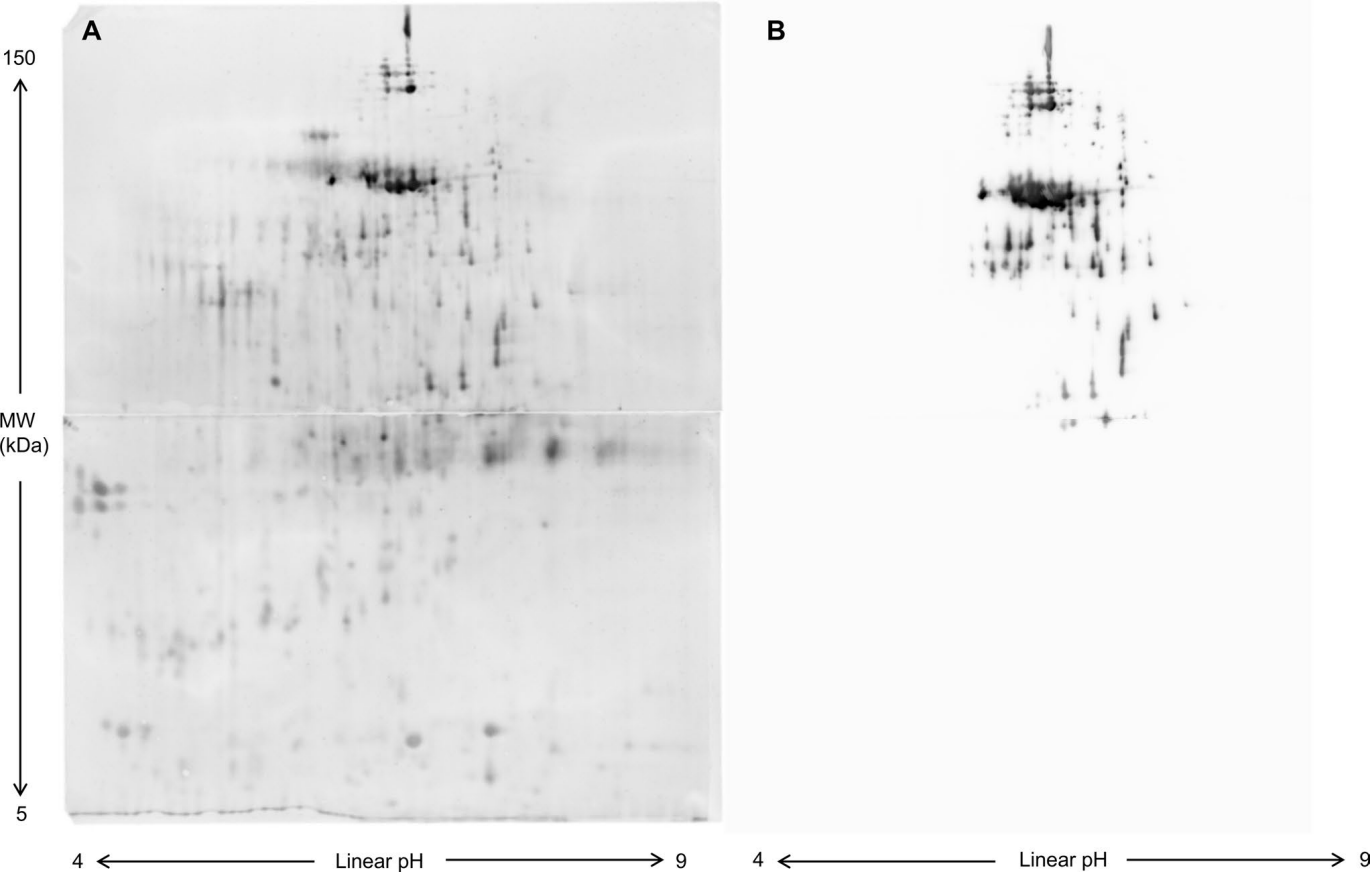
Two-dimensional WB of salivary proteins to specifically detect AMY1. (**A**) A representative nitrocellulose showing the 2-DE protein map of salivary proteins. Proteins were firstly separated according to pI on Immobiline Dry-Strips (18 cm, linear gradient pH 3–10) and then according to molecular weight on 11-16.5% gradient polyacrylamide gels. Then, proteins were transferred onto nitrocellulose membrane and immediately after blot, the membrane was stained with RuBP. (**B**) The same membrane after detection of the immunoreactive spots using the anti-AMY1 antibody

Circular heat maps of exclusive protein spots in the Pre-Ov and Post-Ov group at different task times are shown in Fig. 2B and C while complete lists of identified proteins are reported in Tables S3 and S4. A significant dysregulation of cystatin proteins (CST1, CST2, CST4, and CSTB) was evidenced in the Pre-Ov group, with various expression changes at different task times. On the other hand, the expression of the immunoglobulin heavy constant α1chain (IGHA1) significantly decreased during all task times. Also, the 14-3-3 proteins were significantly increased in the stress phase (T3) followed by a return to T2 level in the next 20 min (T4). In the Post-Ov group, increased AMY1 11 spots across the task was found. A validation of AMY1 spots was performed by 2-D WB using a specific antibody (Fig. 3). To note down that the presence of several fragments and isoforms of AMY1 in human saliva has been documented [25]. Significant changes in the expression of polymeric immunoglobulin receptor (PIGR), prolactin-inducible protein (PIP), PFN1, IGLC, IGKC, JCHAIN and IGHA1 were also observed. Specifically, for all the spots identified as PIGR, there was a decrease at T3 compared to T2 and an increase at T4 compared to T3, but without reaching the T2 level. As for PIP, there was a decrease at T3 compared to T2 and an increase towards T2 levels at T4 in three spots. In the other spot, there was an increase at T3 compared to T2, followed by a decrease at T4 close to the T2 level. Considering PFN1, IGLC and IGKC, a progressive decrease in their expression was found from T2 to T4. The two spots relative to JCHAIN displayed different behavior. In fact, in one spot a similar decrease was found at T3 and T4 compared to T2, while in the other one the protein level decreased only at T4 compared to both T2 and T3. Finally, for IGHA1, there was an increase at T3 followed by a decrease with the level at T4 lower than at T2.

### Western blot analysis

The expression of stress biomarkers AMY1 and IGHA1 was validated by WB analysis. Nitrocellulose images of 1D salivary protein samples detected by RuBP staining and the uncropped images of western blot analysis were presented in the Supplementary material (Figures S3-S6).

**Fig. 4:**
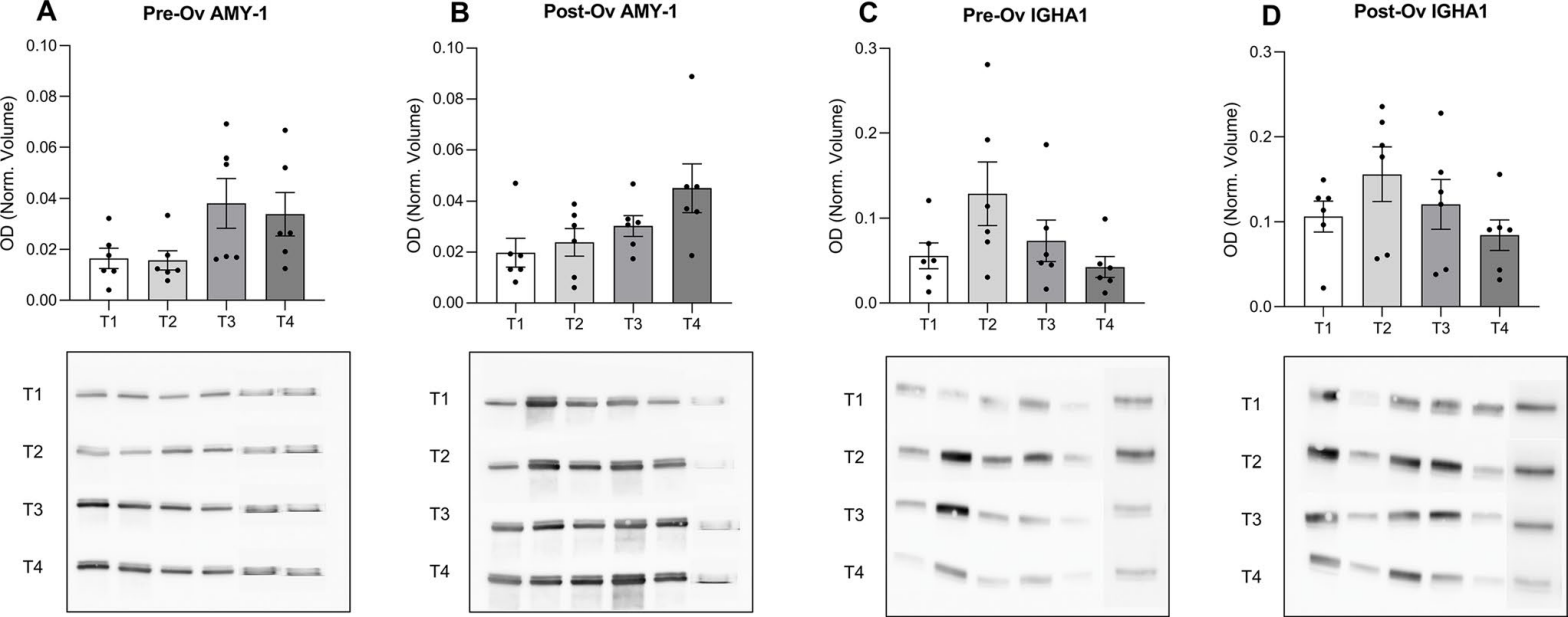
Western blots of salivary AMY1 and IGHA1 in the Pre-Ov and Post-Ov group at different times of the stress task. Histograms of the optical densities of immunoreactive bands (Top) normalized on the total protein content obtained by RuBP staining and representative images (Bottom) of the immunoreactive bands at different times. **A** and **B** panels show the results for AMY1 in the Pre-Ov and Post-Ov group, respectively. **C** and **D** panels represent the results obtained for IGHA1 in the Pre-Ov and Post-Ov group, respectively. ANOVArm was used to compare differences among groups, significance was assessed with a *p*-value < 0.05

Figure 4A, B, C and D summarize the results obtained in two groups at different times of stress task. As far as AMY1 is concerned, its expression seems to be increased throughout the experimental session in both groups (Fig. 4A and B). However, ANOVArm revealed a significant phase effect in the Pre-Ov group (F(3, 15) = 4.79, p-value = 0.0156) and a non-significant increasing trend in the Post-Ov group (F(3, 15) = 3.19, p-value = 0.0543). Moreover, the profiles of amylase increments in the two groups were not superimposable.

For IGHA1, ANOVArm revealed a significant phase effect in both groups (Pre-Ov group (F(3, 15) = 4.41, *p*-value = 0.0205); Post-Ov group (F(3, 15) = 4.88, *p*-value = 0.0146). Specifically, a 2.3-fold and 1.47-fold increase in expression levels was observed at T2 compared to T1 in the Pre-Ov and Post-Ov group, followed by a decrease at T4 with values comparable to T1 (Fig. 4C and D). Comparison between groups at all times showed higher average levels of IGHA1 in the Post-Ov than Pre-Ov group. This finding agrees with reported low levels of immunity components in uterine secretions during the follicular phase [26].

### Ingenuity pathway analysis

**Fig. 5:**
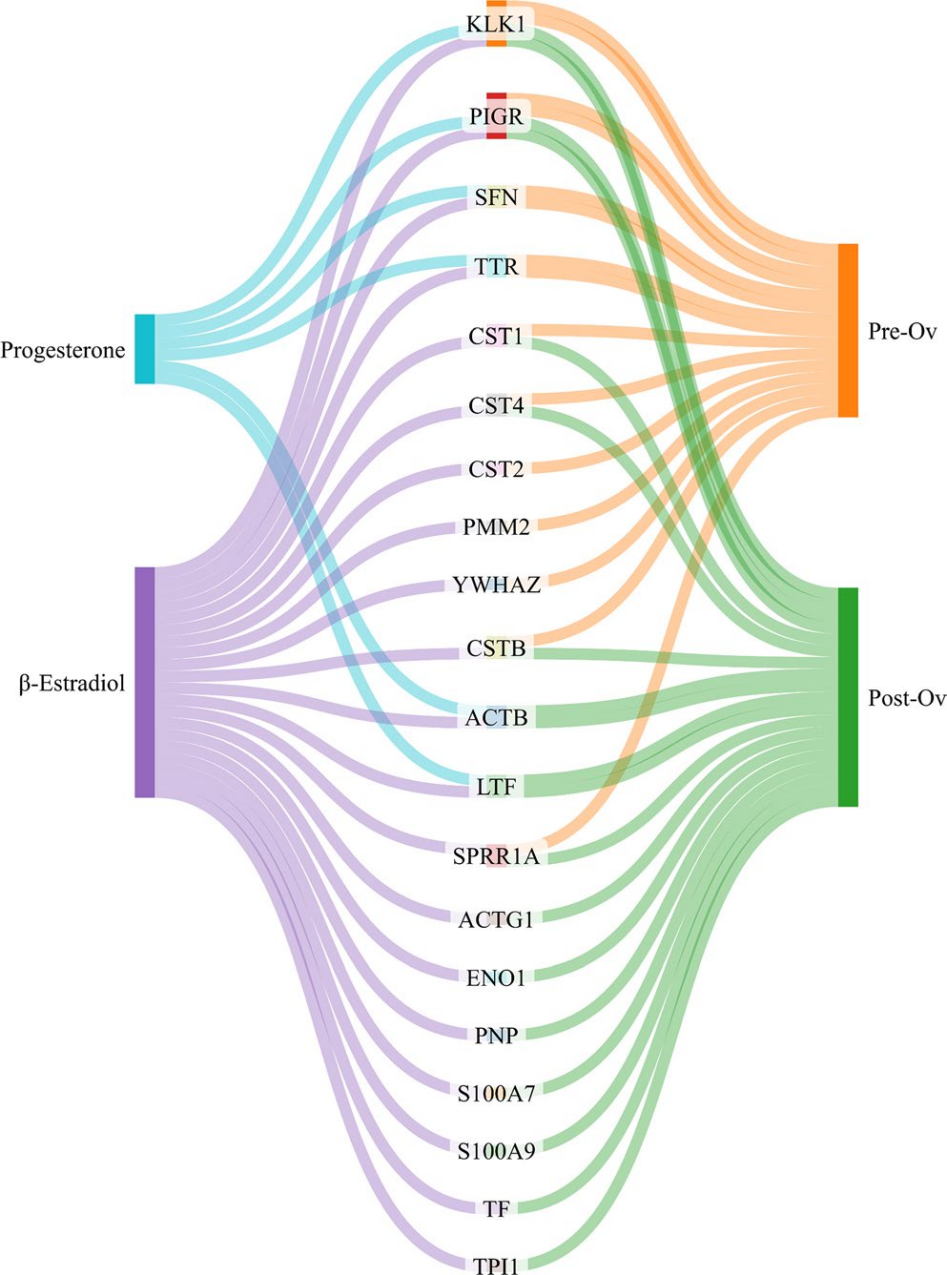
Sankey diagram according to IPA. Proteins found differentially expressed in the Pre-Ov and Post-Ov group were analysed by IPA to predict upstream regulators with significant *z*-score. Both progesterone and β-estradiol were found as upstream regulators but only β-estradiol reached significant positive *z*-score (> 2) in the Pre-Ov group. In the middle of graph, each protein contributing to predict the potential upstream regulators is identified by the corresponding gene name

The differentially expressed proteins, related to each group, were analysed by means of IPA to recognize their molecular and cellular functions and investigate upstream regulators. Based on differentially expressed proteins, IPA analysis suggested progesterone and β-estradiol as predicted upstream regulators Sankey diagram (Fig. 5) represents the association between deregulated proteins and the regulators in the Pre-Ov and Post-Ov group. Positive *z*-score value (> 2) was found for β-estradiol in Pre-OV group whereas progesterone did not reach a significant threshold (*z*-score value of 1.07).

### Correlation between ovarian hormones and protein spots

**Fig. 6:**
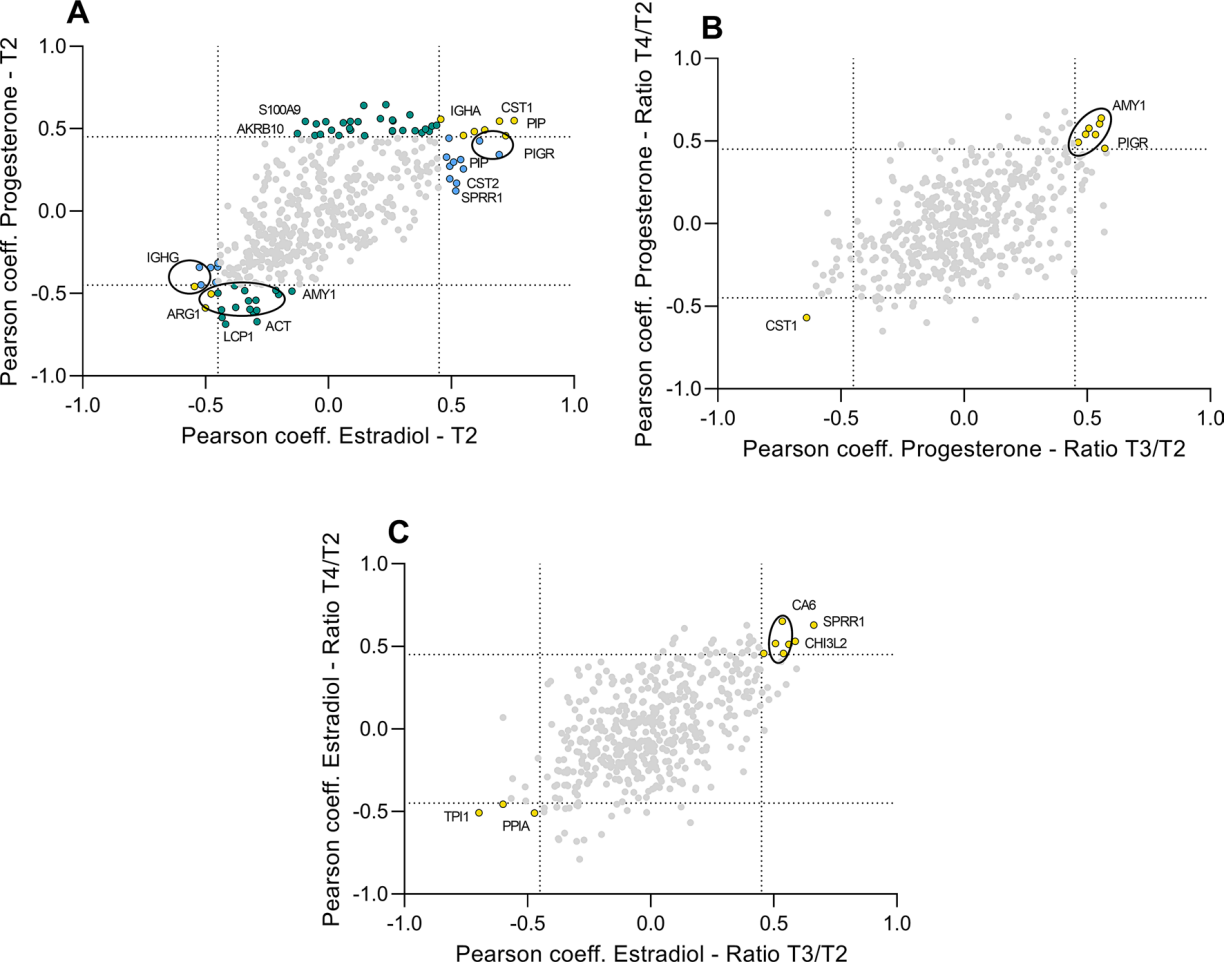
Graphical representation of Pearson coefficients derived from six different bivariate Pearson correlations between hormones and protein spots. (**A**) The graph represents the correlation of Pearson coefficient values obtained from β-estradiol-T2 correlation (X-axis) and progesterone-T2 correlation (Y-axis) (Table S6; S7). (**B**) The graph shows the correlation of Pearson coefficient values derived from progesterone-T3/T2 correlation (X-axis) and progesterone-T4/T2 correlation (Y-axis) (Table S8). (**C**) The Graph represents the correlation of Pearson coefficient values obtained from β-estradiol-T3/T2 correlation (X-axis) and β-estradiol-T4/T2 correlation (Y-axis) (Table S9). In all panels dashed lines represent the threshold value of Pearson coefficient, set as ± 0.45. Green (only T2 and progesterone correlation), blue (only T2 and β-estradiol correlation), and yellow (both correlations) stained circles represent significant protein spots labelled using the gene names. Spots with the same identification are encircled

Bivariate Pearson correlation between OH levels and normalized optical density of protein spots at T2 revealed 36 and 31 spots correlating with progesterone and β-estradiol, respectively. Twelve of these spots correlated with both hormone levels (Fig. 6A, Tables S5 and S6). Notably, a positive correlation between progesterone levels and PIGR and a negative one with actin cytoplasmic 1 (ACTB) was found. Moreover, a positive correlation between β-estradiol levels and cystatin proteins (CST1, CST2, CST4), triosephosphate isomerase 1 (TP1), cornifin-A (SPRR1A), 14-3-3 protein zeta/delta (YWHAZ) and PIGR was observed.

In order to assess whether spot density changes across the anxiety task were associated with OH concentrations we carried out a correlation analysis between hormone concentrations and T3/T2 and T4/T2 ratio of the protein spots considering only spots, which showed a significant correlation with both ratios. Especially, 8 and 11 protein spots generated a positive correlation with progesterone and β-estradiol, respectively (Fig. 6B and C, Tables S7 and S8).

It is intriguing to observe numerous spots of AMY1 correlated with progesterone at both T2 and ratios. Simultaneously, we noted a correlation between β-estradiol and an increment of CA6 while this protein did not correlate during the relaxation phase.

## Discussion

The main aim of this study was to investigate the effect of OH on salivary protein secretion induced by test anxiety. Women either in the mid-luteal phase (Post-Ov group) or in the early follicular phase (Pre-Ov group) of their menstrual cycle participated in the simulation of an oral examination and saliva samples were collected before and after the task. Analysis of salivary β-estradiol and progesterone concentrations revealed higher hormonal levels in the Post-Ov than in the Pre-Ov group, which is consistent with the expected hormonal difference between the two phases of the period. The use of salivary sex hormone monitoring as a feasible and accurate method of tracking menstrual cycle phases is supported by previous evidence of strong correlations between salivary and blood concentrations of estradiol and progesterone throughout the menstrual cycle in young premenopausal women [27].

Analysis of self-reports indicated medium levels of social and general anxiety in both groups. This finding is consistent with a previous study with university students [18] and is not surprising given that moderate levels of anxiety are common among students who frequently encounter demanding circumstances such as the pressure associated with examinations that are unavoidable sources of anxiety [28]. The similarity of the groups’ scores on the various tests also rules out the presence of baseline differences in general and social anxiety between the two groups.

STAY-Y1 results indicated that the simulation was effective in inducing test anxiety. In fact, in all participants, perceived anxiety was significantly higher after the task than in the post-relaxation period. This finding confirms the results of previous studies that applied the same experimental protocol [9, 18].

The anxiety task effectiveness was also confirmed by PREP scores, which showed high levels of nervousness and low levels of calm in all participants. Interestingly, the Post-Ov group reported higher scores for Confidence and Preparedness and higher performance expectations than the Pre-Ov group suggesting that it is the cognitive dimension of test anxiety, rather than the emotional dimension, that may be modulated by OH. This finding accords with recent evidence indicating that the relationship between worry and cognitive control is sensitive to OH mean levels [29].

The task efficacy was also confirmed by the results of RR intervals analysis. In fact, in all participants, RR shortened significantly throughout the task, with the lowest values during the oral presentation, indicating an anxiety-related activation of the sympathetic nervous system (SNS).

Differential expression of salivary proteins between Pre-Ov and Post-Ov groups was found both after relaxation (T2) and at different times following the test-anxiety task (T3, T4).

Previous evidence has shown that both acute mental and physical stress are able to induce marked changes in salivary protein secretion with a short latency (within 5–10 min) from the onset of stress [9, 30] so they could be considered biomarkers of acute stressful events. However, in the present study, we also collected a saliva sample 20 min after the end of the task (T4) to avoid excluding proteins whose secretion changes with a longer latency and to better follow the time course of proteins that change with a shorter latency.

With regard to the possibility that test anxiety might affect protein concentration by altering salivary flow rate, an interesting study using an acute mental stress, namely a two-minute oral speech, showed that regardless of perceived anxiety and the increase in cortisol secretion, there was no effect of the task on salivary flow rate, either immediately after the end of the task or at later times [31]. However, as other factors such as age, sex, circadian rhythms and mechanical/chemical stimulation of the oral cavity could affect salivary flow rate, our participants were of similar sex and age, saliva samples were collected at approximately the same time of day and no oral cavity stimuli were administered. In addition, we collected the same amount of saliva from all participants and all were able to provide the specified amount of saliva (1 ml) within approximately 1 min.

### Relaxation: T2 vs. T2

Following relaxation, in the Post-Ov group IGLC, IGKC, and JCHAIN levels (2-DE) appear higher than the Pre-Ov group and even though these differences do not reach the statistical significance, correlation analysis indicated a positive association between both progesterone and β-estradiol and these immunoglobulin chains. Previous research has shown that reduced levels of salivary free light chains and IgA can be considered biomarkers of the negative impact of psychological stress on mucosal immunity [32]. So, our findings suggest that OH might favour the effectiveness of relaxation, in terms of both reducing psychological stress and positively modulating oral mucosa immunity. Negative correlations between the density of AMY1 spots and both OH were also found. As a higher salivary AMY1 concentration is considered a marker of stress and anxiety, these findings corroborate the hypothesis suggested by immunoglobulin results that higher levels of OH may improve the effectiveness of relaxation techniques in reducing stress.

### Task: common proteins expression

Twenty-nine spots significantly changed across the task in both groups, representing proteins whose concentration varies under anxiety conditions independently of the OH influence. As expected, a significant increase of spots identified as AMY1 was observed across the task in both groups. This finding confirms the efficacy of the experimental protocol in inducing test anxiety. In fact, α-amylase is considered an indirect indicator of SNS activity and is sensitive to both acute and chronic psychophysiological stressors [9, 26, 33]. However, WB analysis revealed a marked surge in the Pre-Ov group immediately following the task conclusion, while a more gradual non-significant increase was found in the Post-Ov group suggesting a potential negative modulation of the SNS activity by OH.

A reduction in spots identified as immunoglobulins light and J chains was found in both groups.

Changes in free immunoglobulin light chains, that are secreted in saliva by local plasma cells and whose secretion rate correlates with IgA [34], have been described in relation to both acute and prolonged physical stress [35] and chronic psychosocial stress (e.g. university examinations) [32]. Specifically, their secretion rates were significantly lower at the exam period compared to baseline. However, no association with increased vulnerability to infections and/ or reduced well-being has been reported [32].

Two-DE did not reveal significant changes in common spots identified as IgA, even though WB analysis revealed a similar decrease in both groups across the task, with slightly higher IgA levels in the Post-Ov than Pre-Ove group. This suggests that OH may reduce the negative impact of psychological stress on mucosal immunity.

Among the common proteins there are CA6 and CST4. CA6 is the only secretory isoenzyme of the mammalian CA gene family [36], it is exclusively expressed in the serous acinar cells of the parotid and submandibular glands and is then secreted into saliva. Previous evidence suggested that α-amylase and CA6 are similarly regulated by the autonomic nervous system and may be co-released [37]. A recent study has indicated that CA6 may be elevated in response to physical stress [38]. However, there is currently lack of evidence suggesting this is also the case for psychological stress. CST4 levels increased after the task and returned near to the pre-task levels after 20 min from the task completion. This is in accord with previous literature, which has identified cystatins as potential markers of acute stress [24].

### Exclusive proteins

A markedly high number of spots was found in the Post-Ov group and not in the Pre-Ov group. This discrepancy could be attributed to the potential modulation of specific stress-related proteins by OH, either directly or indirectly.

Some spots exclusive of the Pre-Ov group were identified as the protease inhibitors cystatins (CST1, CST2, CST4, and CSTB). The transient increase of CST1, CST2 and CST4, immediately following the task, is consistent with previous literature, which has identified cystatins as potential markers of acute stress [24]. In contrast, a reduction in CSTB, an endogenous cathepsin inhibitor that is present in various cell types and extracellular fluids, involved in diverse cellular processes (e.g., apoptosis prevention) and regarded as a component of the innate immune system [38–40] was observed 20 min following task conclusion. To date, the exact function of salivary CSTB is unclear [42] and there is no available data about the possible function of a stress-induced increase in salivary cystatins levels nor about their modulation by OH.

Another exclusive protein of the Pre-Ov group exhibiting a transient increase immediately after task conclusion is YWHAZ. Previous studies have shown that 14-3-3 proteins are found primarily in neurons and may activate hydroxylation of tyrosine and tryptophan, which is critical for the synthesis of dopamine and other neurotransmitters [43]. Furthermore, the importance of the 14-3-3 protein in the regulation of synaptic functions and neuron plasticity has been demonstrated [44]. However, to date, no information is available regarding the potential role of salivary 14-3-3 proteins in psychosocial anxiety.

Eleven spots, which increased exclusively in the Post-Ov group, were identified as AMY1 isoforms and fragments. Different forms of AMY1in human saliva have been categorized as isobaric, truncated and alternative α-amylases [25, 45]. In both the isobaric and alternative forms, the catalytic sites remain unchanged indicating the maintenance of their hydrolytic activity. The specific mechanisms by which AMY1 is modified are yet to be elucidated. Nevertheless, it is possible to speculate that OH may facilitate post-translation modifications prior to AMY1 secretion in the oral cavity.

Many exclusive spots of the Post-Ov group and identified as immunoglobulin light and J chains showed a reduction across the task. This behaviour was superimposable to that described for the light and J chain common spots. Moreover, 1 (Pre-Ov) and 2 (Post-Ov) spots identified as IGHA1 showed significant but modest density variation during the task even though the profiles were slightly different in the two groups. Anyway, this finding agrees with WB results. A decrease was also found for the spot identified as PIGR, which is considered an indicator of immunoglobulins glandular transport capacity [46]. This similarity suggests that the immunoglobulin secretion decrease is likely due to a reduction in glandular transport capacity rather than a reduced release of IgA from B lymphocytes.

Immediately after the task, the slight density reduction of 2 spots, identified as PIP, in the Post-Ov group is consistent with our previous results [9] but it contrasts with another study in which the Trier Social Stress Test was used [24]. However, these studies included both males and females, and no consideration was given to the phase of the menstrual cycle or the OH levels. Interestingly, after 20 min from the task conclusion, PIP levels increased again reaching higher values than those found after relaxation. As PIP plays a role in oral cavity immunity including anti-inflammatory effects and inhibition of bacterial growth [47], our results may suggest a protective role of OH, which manifests itself with some latency after the end of the stressful task.

Another exclusive spot was identified as PFN1. PFN1 levels, similarly to immunoglobulin light and J chains, were higher after relaxation in the Post-Ov group than the Pre-Ov group and significantly decreased across the task in the Post-Ov group. This suggests a similar modulation of their secretion by OH. Previous studies have demonstrated a relation between PFN1 levels and ovarian function [48], however the relationship between PFN1 and psychophysiological anxiety has not been previously investigated.

The possible modulation of salivary proteins by OH was suggested by IPA, which particularly indicated β-estradiol as the main upstream regulator of the proteins whose expression was dysregulated by test anxiety and by the significant correlations between both OH levels and many of the differentially expressed proteins in the two groups.

Overall, our results provide preliminary evidence that the menstrual cycle may be a significant driving factor for differences in the subjective and physiological responses prompted by test anxiety. In particular, the study suggests that OH might play a role in enhancing the oral mucosa immunity during relaxation, and presents preliminary evidence indicating that OH may also mitigate the adverse effects of psychological stress on mucosal immunity. Although the results are encouraging, further validation is required on a larger sample.

Furthermore, in the present study, two distinct groups of participants were recruited due to the inherent limitations of the experimental protocol, which precludes the use of a within-subject design. Indeed, at the end of the task, a debriefing session was held with the participants to help them understand the real purpose of the study, with the intention of promoting recovery. Since the participants were informed that the “exam” was a fake, this made it impossible for them to take part in a second experimental session. However, given the considerable inter-cycle and inter-subject variability in ovarian hormone levels, it would be advisable to use a within-subject design, i.e. an experimental protocol in which each participant is tested twice, specifically during the early follicular and mid-luteal phases of the menstrual cycle.

Moreover, it would be of interest to replicate the experiment with a cohort of young, healthy women who are taking oral contraceptives that effectively abolish the physiological hormonal fluctuations that occur during the menstrual cycle, or with a male cohort as a comparison group.

Also, as some of the salivary proteins differentially expressed in the two groups are involved in oral immunity and other functions such as neuroplasticity and synaptic transmission, but their functions have not previously been associated with either psychophysiological anxiety or ovarian hormones, further studies would be advisable to clarify these new findings.

Finally, given the preliminary evidence on the modulation by OH on the bidirectional brain-heart interactions, which suggest increased brain-to-heart interplay involving the vagal activity limited to the Post-Ov group and a positive correlation between progesterone levels and brain-to-heart indices in all the EEG bands, at midline-right frontal and central brain areas [49], further studies will be conducted on a larger sample of participants to investigate the possible associations between ovarian hormones levels, salivary proteome, BHI measures and scores of perceived test anxiety.

## Electronic supplementary material

Below is the link to the electronic supplementary material.

Supplementary Material 1

Supplementary Material 2

## Data Availability

Raw data files are available from the corresponding author upon reasonable request.
