# Supplementary material for: Modulation of test anxiety-induced salivary protein secretion by ovarian steroid hormones: a preliminary study
Source: J Physiol Biochem. 2025 Feb 8;81(1):215–28. doi: 10.1007/s13105-025-01067-w (PMC11958418; doi:10.1007/s13105-025-01067-w)
Supplement: Supplementary file 2 — Supplementary Material 2 [file 13105_2025_1067_MOESM2_ESM.pptx]

## Slide 1
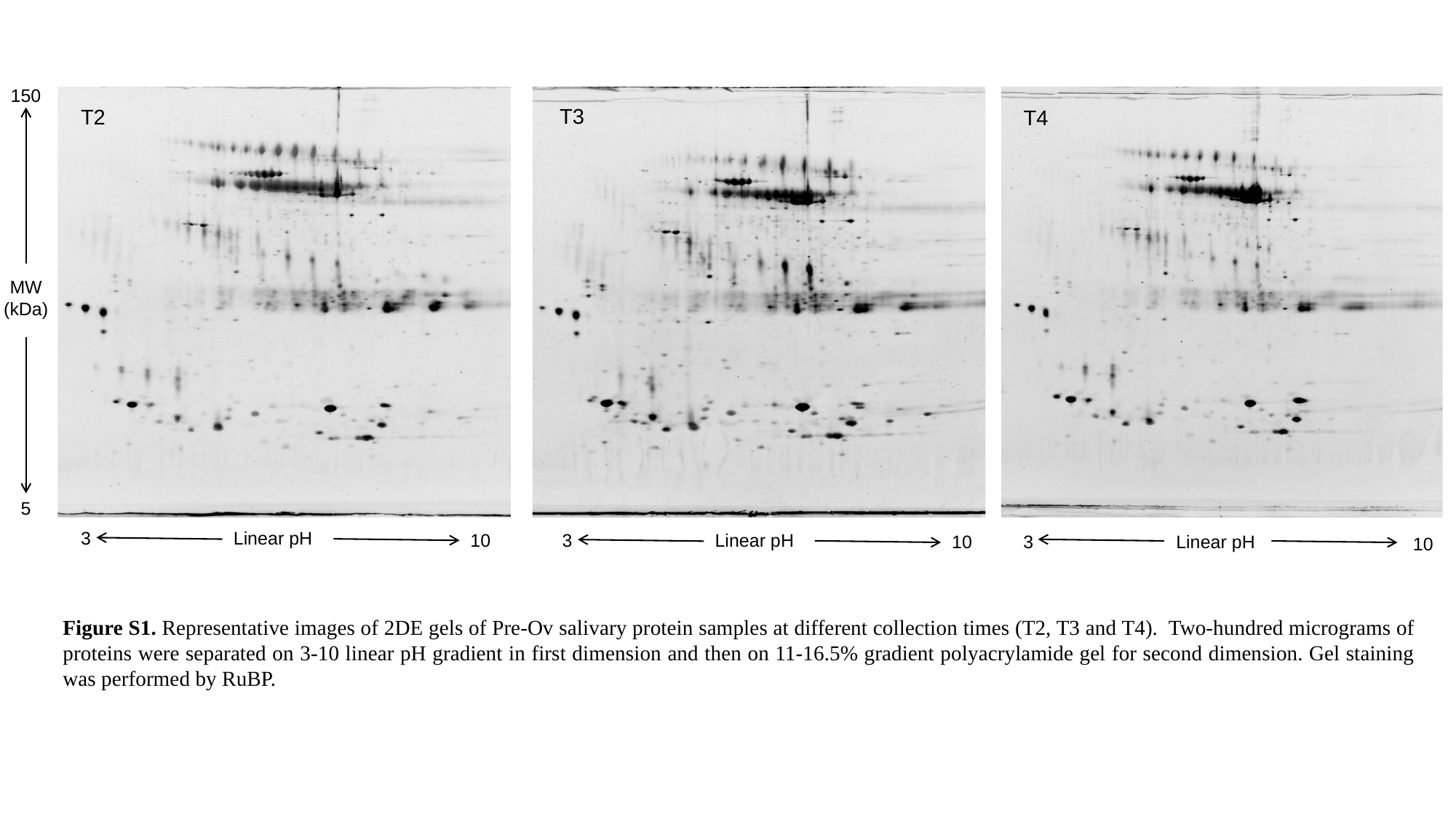

150
MW
(kDa)
5
T3
T2
T4
3
Linear pH
10
3
Linear pH
10
3
Linear pH
10
Figure S1. Representative images of 2DE gels of Pre-Ov salivary protein samples at different collection times (T2, T3 and T4). Two-hundred micrograms of proteins were separated on 3-10 linear pH gradient in first dimension and then on 11-16.5% gradient polyacrylamide gel for second dimension. Gel staining was performed by RuBP.

## Slide 2
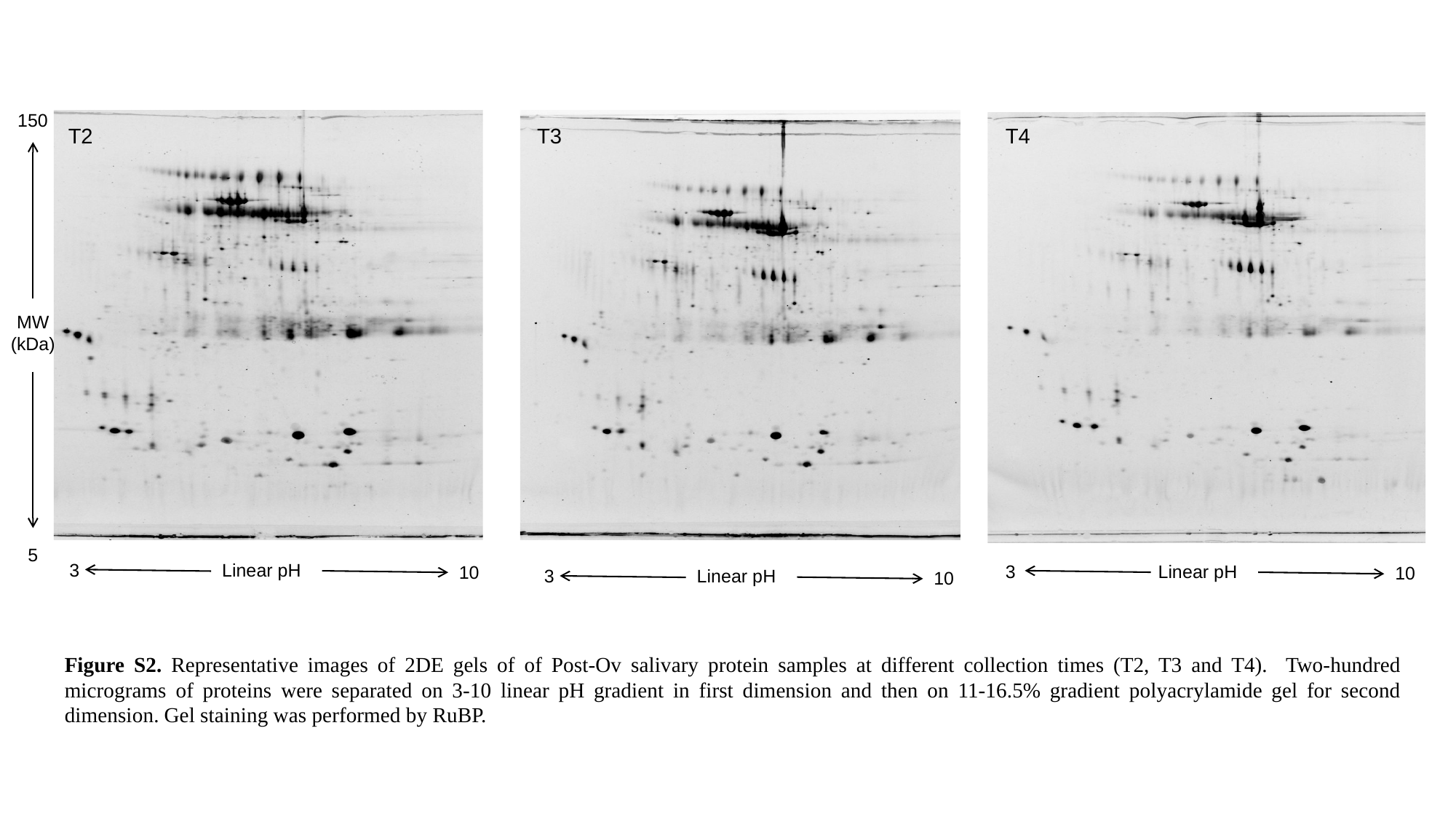

150
MW
(kDa)
5
T4
T3
T2
3
Linear pH
10
3
Linear pH
10
3
Linear pH
10
Figure S2. Representative images of 2DE gels of of Post-Ov salivary protein samples at different collection times (T2, T3 and T4). Two-hundred micrograms of proteins were separated on 3-10 linear pH gradient in first dimension and then on 11-16.5% gradient polyacrylamide gel for second dimension. Gel staining was performed by RuBP.

## Slide 3
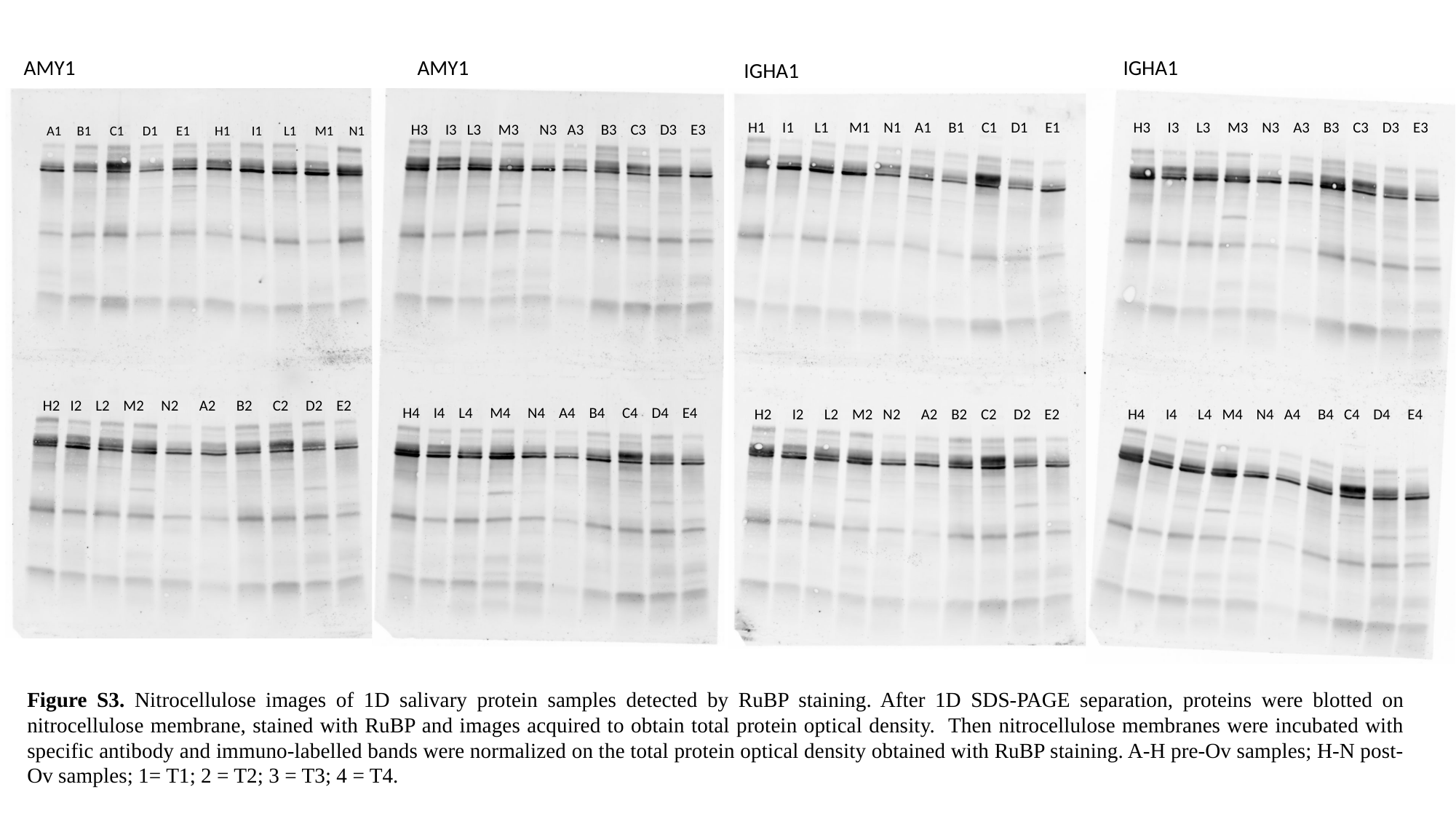

AMY1
IGHA1
AMY1
IGHA1
H1 I1 L1 M1 N1 A1 B1 C1 D1 E1
H3 I3 L3 M3 N3 A3 B3 C3 D3 E3
H3 I3 L3 M3 N3 A3 B3 C3 D3 E3
A1 B1 C1 D1 E1 H1 I1 L1 M1 N1
H2 I2 L2 M2 N2 A2 B2 C2 D2 E2
H4 I4 L4 M4 N4 A4 B4 C4 D4 E4
H2 I2 L2 M2 N2 A2 B2 C2 D2 E2
H4 I4 L4 M4 N4 A4 B4 C4 D4 E4
Figure S3. Nitrocellulose images of 1D salivary protein samples detected by RuBP staining. After 1D SDS-PAGE separation, proteins were blotted on nitrocellulose membrane, stained with RuBP and images acquired to obtain total protein optical density. Then nitrocellulose membranes were incubated with specific antibody and immuno-labelled bands were normalized on the total protein optical density obtained with RuBP staining. A-H pre-Ov samples; H-N post-Ov samples; 1= T1; 2 = T2; 3 = T3; 4 = T4.

## Slide 4
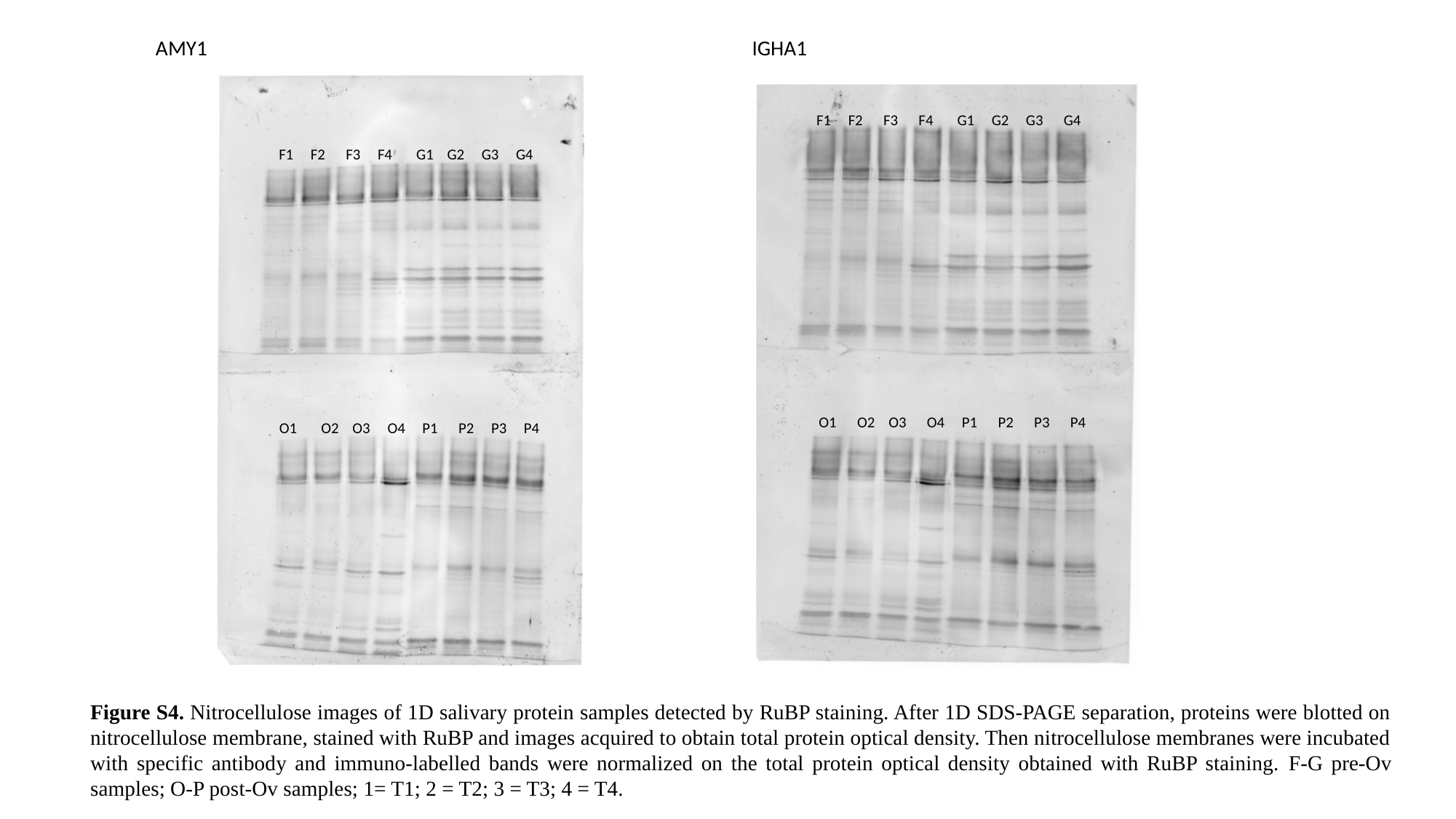

AMY1
IGHA1
F1 F2 F3 F4 G1 G2 G3 G4
O1 O2 O3 O4 P1 P2 P3 P4
F1 F2 F3 F4 G1 G2 G3 G4
O1 O2 O3 O4 P1 P2 P3 P4
Figure S4. Nitrocellulose images of 1D salivary protein samples detected by RuBP staining. After 1D SDS-PAGE separation, proteins were blotted on nitrocellulose membrane, stained with RuBP and images acquired to obtain total protein optical density. Then nitrocellulose membranes were incubated with specific antibody and immuno-labelled bands were normalized on the total protein optical density obtained with RuBP staining. F-G pre-Ov samples; O-P post-Ov samples; 1= T1; 2 = T2; 3 = T3; 4 = T4.

## Slide 5
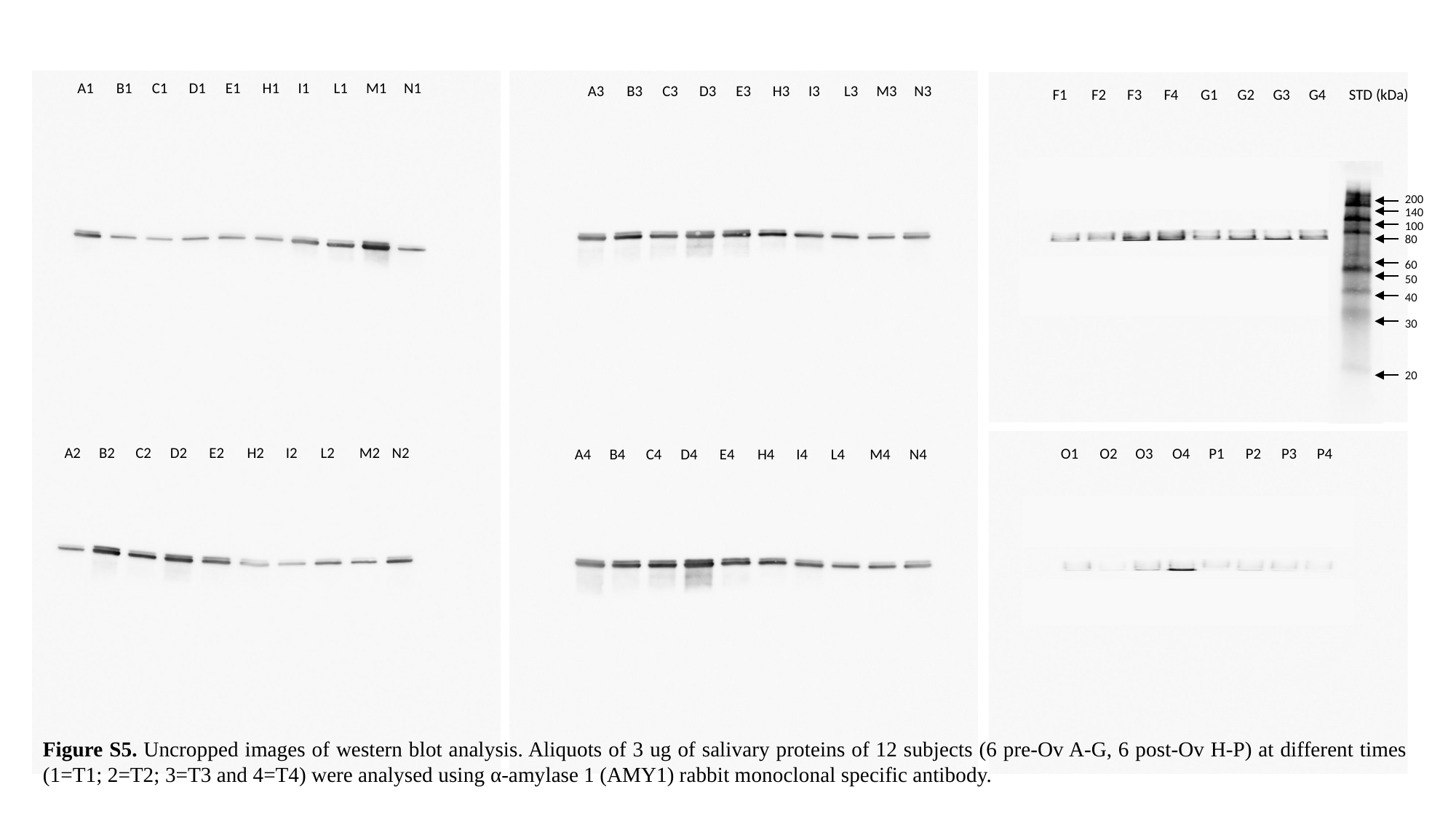

A1
B1
C1
D1
E1
H1
I1
L1
M1
N1
A3
B3
C3
D3
E3
H3
I3
L3
M3
N3
STD (kDa)
F1
F2
F3
F4
G1
G2
G3
G4
200
140
100
80
60
50
40
30
20
A2
B2
C2
D2
E2
H2
I2
L2
M2
N2
O1
O2
O3
O4
P1
P2
P3
P4
A4
B4
C4
D4
E4
H4
I4
L4
M4
N4
Figure S5. Uncropped images of western blot analysis. Aliquots of 3 ug of salivary proteins of 12 subjects (6 pre-Ov A-G, 6 post-Ov H-P) at different times (1=T1; 2=T2; 3=T3 and 4=T4) were analysed using α-amylase 1 (AMY1) rabbit monoclonal specific antibody.

## Slide 6
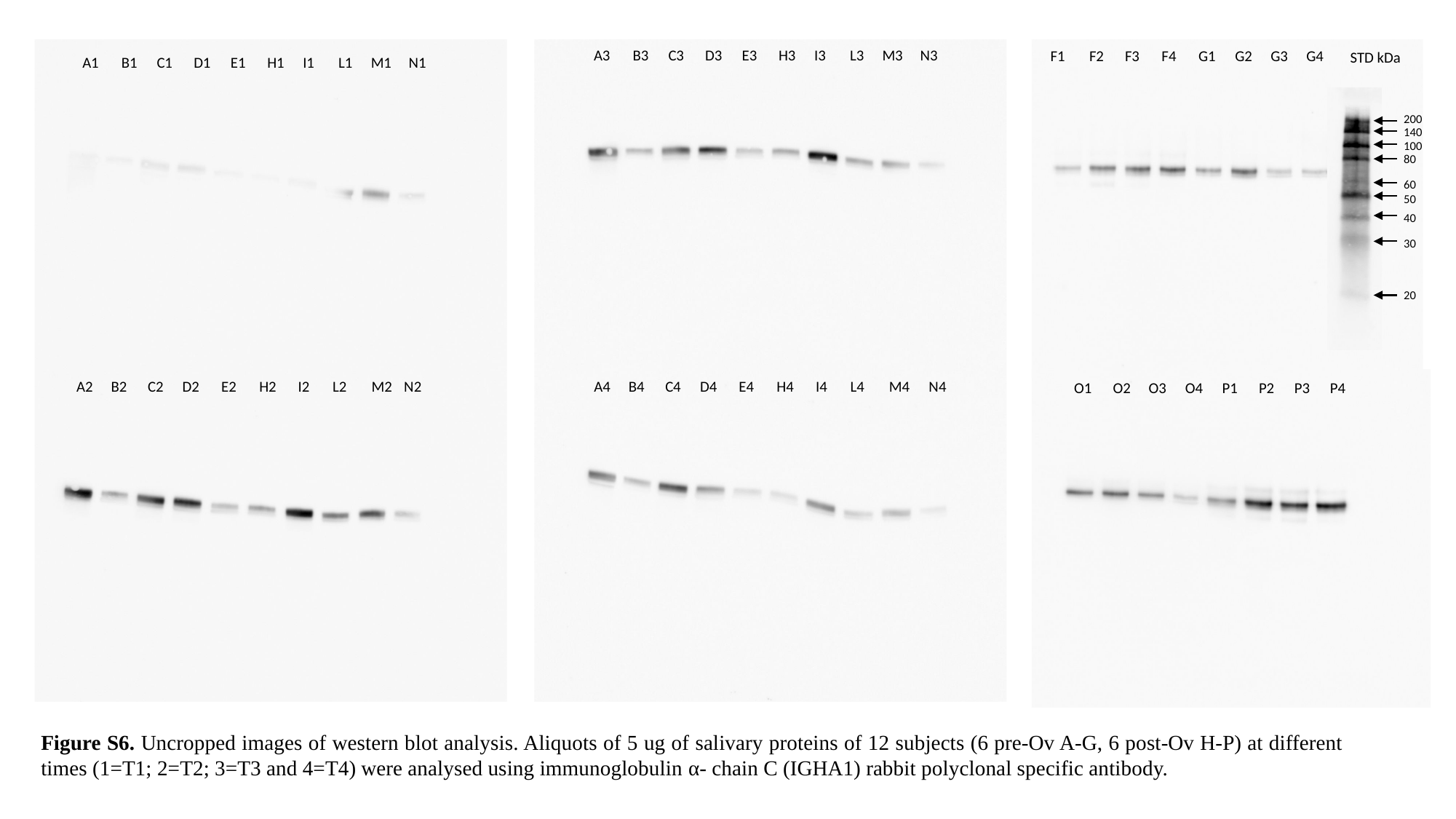

A3
B3
C3
D3
E3
H3
I3
L3
M3
N3
F1
F2
F3
F4
G1
G2
G3
G4
STD kDa
A1
B1
C1
D1
E1
H1
I1
L1
M1
N1
200
140
100
80
60
50
40
30
20
A4
B4
C4
D4
E4
H4
I4
L4
M4
N4
A2
B2
C2
D2
E2
H2
I2
L2
M2
N2
O1
O2
O3
O4
P1
P2
P3
P4
Figure S6. Uncropped images of western blot analysis. Aliquots of 5 ug of salivary proteins of 12 subjects (6 pre-Ov A-G, 6 post-Ov H-P) at different times (1=T1; 2=T2; 3=T3 and 4=T4) were analysed using immunoglobulin α- chain C (IGHA1) rabbit polyclonal specific antibody.
